# Designing an intervention to improve sexual health service use among university undergraduate students: a mixed methods study guided by the behaviour change wheel

**DOI:** 10.1186/s12889-019-8059-4

**Published:** 2019-12-26

**Authors:** Christine Cassidy, Audrey Steenbeek, Donald Langille, Ruth Martin-Misener, Janet Curran

**Affiliations:** 1 Dalhousie University, School of Nursing, 5869 University Avenue, PO BOX 15000, Halifax, NS B3H 4R2 Canada; 2 Dalhousie University, Department of Community Health and Epidemiology, Halifax, NS Canada

**Keywords:** Behaviour change wheel, Sexual health services, Reproductive health, University students, Mixed methods research, Theoretical domains framework

## Abstract

**Introduction:**

University undergraduate students are within the population at highest risk for acquiring sexually transmitted infections, unplanned pregnancy, and other negative health outcomes. Despite the availability of sexual health services at university health centres, many students delay or avoid seeking care. In this study, we describe how the Behaviour Change Wheel was used as a systematic approach to design an intervention to improve sexual health service use among university undergraduate students.

**Methods:**

This paper describes the intervention development phase of a three-phased, sequential explanatory mixed methods study. Phases one and two included a quantitative and qualitative study that aimed to better understand students’ use of sexual health services. In phase three, we followed the Behaviour Change Wheel to integrate the quantitative and qualitative findings and conduct stakeholder consultation meetings to select intervention strategies, including intervention functions and behaviour change techniques.

**Results:**

Key linkages between opportunity and motivation were found to influence students’ access of sexual health services. Stakeholders identified six intervention functions (education, environmental restructuring, enablement, modelling, persuasion, and incentivization) and 15 behaviour change techniques (information about health consequences, information about social and environmental consequences, feedback on behaviour, feedback on outcomes of behaviour, prompts/cues, self-monitoring of behaviour, adding objects to the environment, goal setting, problem solving, action planning, restructuring the social environment, restructuring the physical environment, demonstration of the behaviour, social support, credible source) as relevant to include in a toolbox of intervention strategies to improve sexual health service use.

**Conclusions:**

This study details the use of the Behaviour Change Wheel to develop an intervention aimed at improving university students’ use of sexual health services. The Behaviour Change Wheel provided a comprehensive framework for integrating multiple sources of data to inform the selection of intervention strategies. Stakeholders can use these strategies to design and implement sexual health service interventions that are feasible within the context of their health centre. Future research is needed to test the effectiveness of the strategies at changing university students’ sexual health behaviour.

## Background

Sexually transmitted infections (STIs) and associated health consequences are of significant concern for young adults. In Canada, young men and women aged 20 to 24 have the highest rates of chlamydia infections (1627.6 per 100,000) [1]. Youth are also at risk of unplanned pregnancy and encounter barriers to accessing effective contraceptive methods [2]. Many university students are among this high-risk group for acquiring STIs and unplanned pregnancy. Effective prevention relies on regular contraceptive use and early detection and treatment [3]. As such, university health centres are essential for preventing negative health outcomes and promoting healthy sexual behaviours among students. Despite students’ risk and the availability of these services, many university students delay or avoid seeking sexual health care. In the United States for example, approximately 27% of college students have ever accessed sexual health services, including: STI, Pap, and pregnancy testing; STI treatment; contraceptive prescriptions; and testicular and gynecological exams [4]. In a Canadian sexual health services study of two universities in Nova Scotia, only 41% of sexually active female students and 25% of male students reported having ever been tested for STIs [5].

Barriers and enablers to sexual health service use include: students’ knowledge and awareness of sexual health services, accessibility of services, peer influence, stigma and feelings of shame, and relationships with health care providers [4, 6]. These barriers and enablers interact with a campus culture that promotes risky behaviours and in turn, influences students’ capability, opportunity, and motivation for accessing sexual health services [6]. As such, targeted interventions are needed to address these barriers and ensure adequate sexual health promotion and illness prevention for students.

**Fig. 1:**
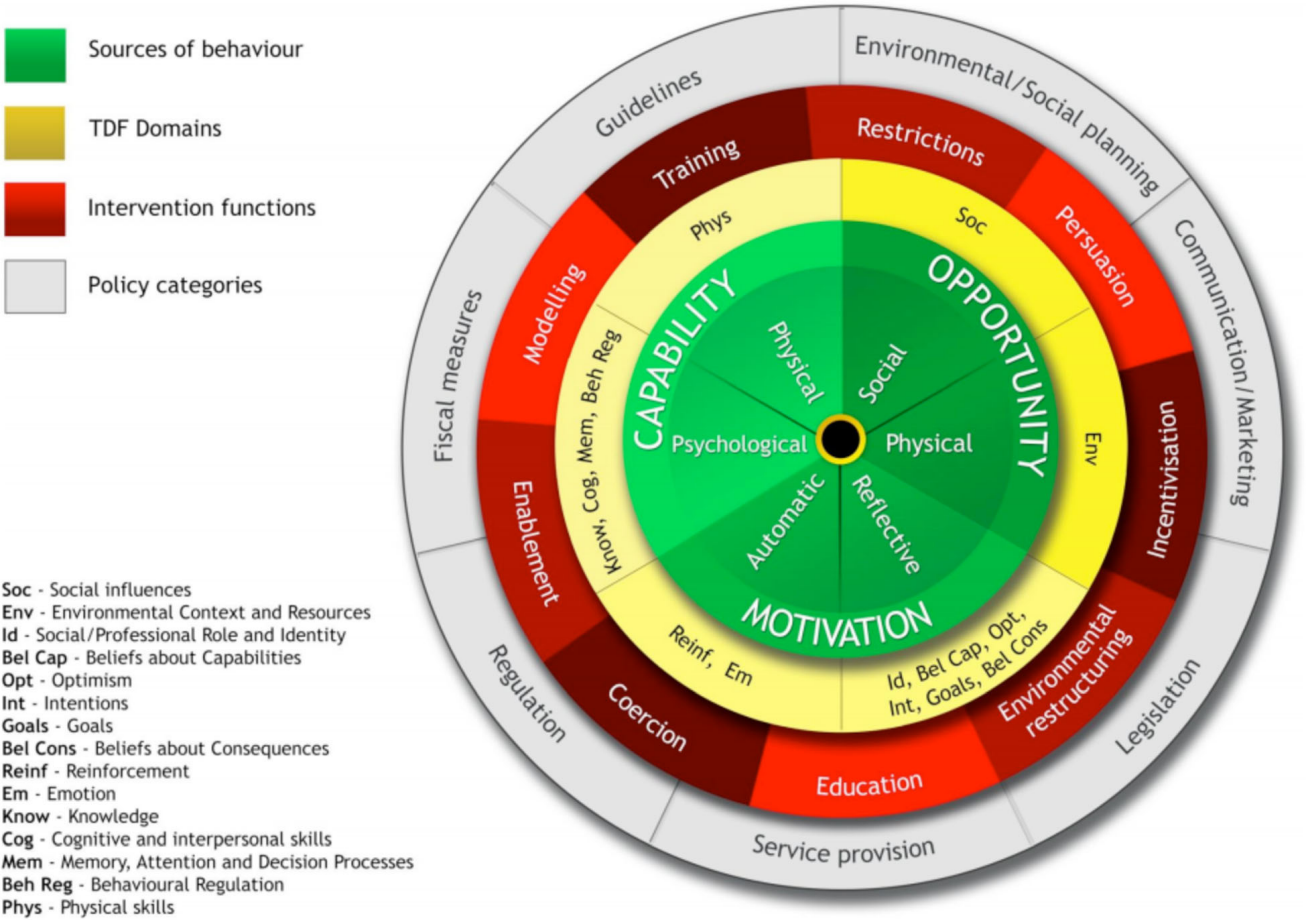
Behaviour Change Wheel [12] (Permission to reproduce from authors and publisher) [12]

Previous studies report positive intervention effects for increasing the uptake of sexual health services [7–9]; however, these behaviour change interventions are poorly described in the published literature [10]. Without a clear description, it is difficult to implement an intervention in the way it was intended and replicate its effects in subsequent research studies. Implementation scientists recommend a systematic, theory-based approach to intervention design to improve development and description [10, 11]. The Behaviour Change Wheel (BCW) is one such approach that offers theory-based tools to help understand and change behaviour (Fig. 1). The BCW is a synthesis of 19 existing behaviour change frameworks and provides a systematic, comprehensive approach to designing interventions. At its core is the COM-B model, which suggests that behaviour change occurs when there is a change in an individual’s capability, opportunity and/or motivation [12]. The Theoretical Domains Framework (TDF) can be used to expand on the COM-B components and provide a more detailed understanding of the behaviours and identify what factors need to be addressed to change behaviour [13]. The BCW identifies nine intervention functions that can be linked to 93 possible behaviour change techniques (BCTs), or “active ingredients” on which to base intervention content [14]. Lastly, the BCW provides guidance on selecting relevant policies and intervention modes of delivery [12]. Studies have used the BCW to guide intervention design in a variety of health care settings, including smoking cessation [15], alcohol reduction [16], condom use [17], and sexual counselling [18].

This paper reports a systematic, theory-based approach to design an intervention to improve sexual health service use among university undergraduate students in Nova Scotia, Canada. The objectives of this final intervention design and description phase were to: 1. Integrate findings from previous phases [5, 6, 19]; 2. Build a toolbox of theory- and evidence-based intervention strategies that can be used to improve the use of sexual health services among university students; and, 3. Describe the utility of the BCW in the area of sexual health service intervention development.

## Methods

**Fig. 2:**
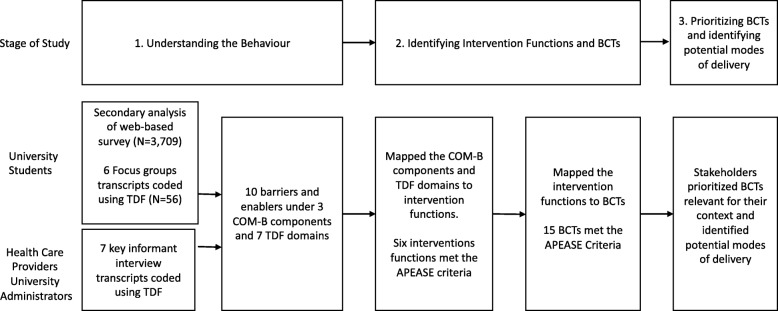
Summary of study stages and intervention content selection

This three-phased study employed a sequential explanatory mixed methods [20] research design guided by the BCW [12] (Fig. 2). Full study methods and Phase 1 and 2 results have been published elsewhere [5, 6, 19]. The final phase described here included stakeholder consultation meetings to identify intervention content.

### Step 1: understand the behaviour

We previously conducted two studies to gain a better understanding of university students’ sexual health service use [5, 6, 19]. The first study involved a secondary analysis of survey data [21] to describe the patterns of sexual health service use among university undergraduate students at two universities in Nova Scotia, Canada [5]. The second study involved focus groups with university undergraduate students, aged 18 to 25, and key informant interviews with health care providers and university administrators at the same two universities to identify barriers and enablers to sexual health service use. The focus group and interview guides and data analysis were guided by the TDF and COM-B model [6]. Following data analysis, we brought the initial themes to a group of students at each university for a member checking exercise which provided participants the opportunity to offer clarification, add information, and prioritize the initial themes [6]. Lastly, the quantitative and qualitative data were integrated using a triangulation protocol [22].

### Step 2: identify intervention content

The research team met to review Phase 1 and 2 findings, identify intervention functions and BCTs, and brainstorm potential modes of intervention delivery. An intervention function is described as a broad category by which an intervention can change behaviour (e.g., education, persuasion, training). The BCW includes a matrix that links each COM-B component and TDF domain to the intervention functions most likely to be effective in bringing about behaviour change [12]. Starting with this matrix, the research team applied the APEASE criteria (affordability, practicability, effectiveness/cost-effectiveness, acceptability, safety, and equity) [ 12] to each intervention function to explore its appropriateness for the sexual health service context.

Next, the research team used the BCT taxonomy (BCTTv1) [14] to identify potential BCTs that would best serve the intervention functions. A BCT is defined as “an observable, replicable, and irreducible component of an intervention designed to alter or redirect causal processes that regulate behaviour” (e.g., demonstration of the behaviour, information about health consequences) [14]. The BCW provides a matrix developed through expert consensus that maps relevant BCTs to intervention functions [12, 23]. Starting with this matrix, the research team used the APEASE criteria to consider which BCTs would be feasible within the context of university sexual health service delivery, and most useful for addressing the identified barriers and enablers to university students’ use of sexual health services. Lastly, to identify potential delivery options, the research team brainstormed modes of delivering each BCT. These were added to a list of modes of delivery developed from the literature review and focus group and interview participant input.

### Step 3: stakeholder consultation

We conducted two stakeholder consultation meetings with health care providers and university administrators at each university to review the findings from Phases 1 and 2 and the intervention content identified by the research team in Step 2. Through discussion, the participants used the APEASE criteria to consider which BCTs would be feasible and prioritized in their university context. Lastly, the participants brainstormed additional modes of delivering each BCT.

## Results

### Step 1: understand the behaviour

**Fig. 3:**
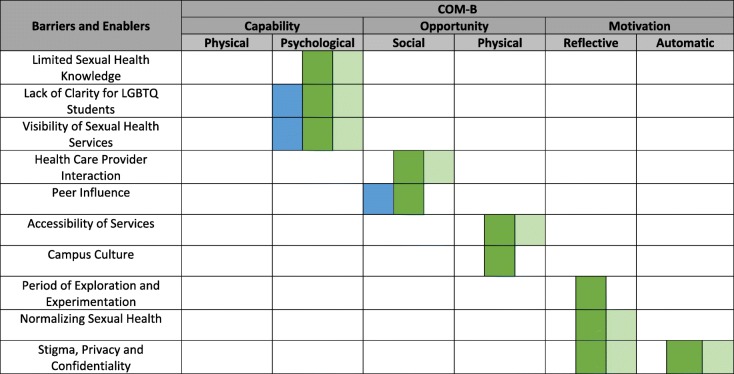
Phase 1 and 2 findings [4, 5] mapped onto COM-B model; *Blue*, phase 1 secondary analysis of online survey; *Dark Green*, phase 2 focus groups with university students; *Light Green*, phase 2 interviews with health care providers and administrators

In phases one and two, we used the COM-B model and TDF to conduct a behavioural assessment of students’ sexual health service use and identified the following COM-B components as important targets: psychological capability, social and physical opportunity, and reflective and automatic motivation (Fig. 3). A summary of the findings from the quantitative and qualitative phases are integrated below. Full study results have been published elsewhere [5, 6].

#### Capability

Both focus groups and interview participants described student’s limited knowledge and awareness of sexual health services as an important barrier to service use. Further, student and health care provider participants identified a lack of understanding on LGBTQ students’ sexual health. Improved visibility of the services was identified as a facilitator to sexual health service use [6].

#### Opportunity

Student participants described physical opportunity, including service accessibility and the campus culture, as both a barrier and enabler to sexual health service use. Due to a campus environment that promotes risky behaviours, student participants described the importance of countering this culture with safe and accessible sexual health services, including flexible hours of operation, convenient location, and mobile clinics (known as STI testing clinics that are offered around campus) [6].

Survey, focus group, and interview data illustrated the importance of peer influence on student behaviour. Participants described the stigma associated with sexual health service use and the influence of peer support on health promotion behaviours. These positive and negative peer influences were found to be important barriers and enablers for accessing sexual health services [5, 6].

#### Motivation

We found that the social influences described above directly affected students’ motivations for accessing sexual health services. Participants stated that accessing the services could jeopardize their privacy and confidentiality and lead to negative emotions (e.g., discomfort, shame, awkwardness). Further, university students are in a developmental period of exploration and experimentation and as a result, felt motivated to access sexual health services while experimenting with high-risk behaviours [6].

#### Contextual differences

While the barriers and enablers to sexual health services were applicable to both universities, we found a number of important contextual differences including: size of student population; clinician knowledge on LGBTQ health; structure of health service delivery; financial resources; and location of services. These contextual elements were important factors to take into consideration when designing interventions for the two universities.

### Step 2: identify intervention content

Following group discussion using the APEASE criteria, the research team identified the following six intervention functions as most useful for addressing the barriers and enablers to sexual health service use among university students: education, environmental restructuring, enablement, modelling, persuasion, and incentivisation (Table 1). University students are the target population for the behaviour change; however, as changing student behaviour requires interaction with both health care providers and the health services, the research team considered the need for multi-level intervention content throughout the development process.

**Table 1:** Barriers and enablers from the COM-B and TDF mapped to intervention functions in the Behaviour Change Wheel

Barriers & Enablers COM-B | TDF	Intervention Functions								
	Education	Persuasion	Incentivisation	Environmental restructuring	Modelling	Enablement	Coercion	Training	Restriction
Limited Sexual Health Knowledge									
Capabilities – Psychological | Knowledge	✔								
Lack of Clarity for LGBTQ Students									
Capabilities – Psychological | Knowledge	✔								
Visibility of Sexual Health Services									
Capabilities – Psychological | Memory, Attention, Decision-Making				✔		✔			
Health Care Provider Interaction									
Opportunity- Social | Social Influences				✔	✔	✔			
Peer Influence									
Opportunity- Social | Social Influences				✔	✔	✔			
Accessibility of Services									
Opportunity – Physical | Environmental Context & Resources				✔		✔			
Campus Culture									
Opportunity – Physical | Environmental Context & Resources				✔		✔			
Period of Exploration and Experimentation									
Motivation – Reflective | Beliefs About Consequences	✔	✔			✔				
Normalizing Sexual Health									
Motivation – Reflective | Optimism	✔	✔			✔	✔			
Stigma, Privacy and Confidentiality									
Motivation – Reflective | Beliefs About Consequences	✔	✔			✔				
Motivation – Automatic | Emotion		✔	✔		✔	✔			

**Fig. 4:**
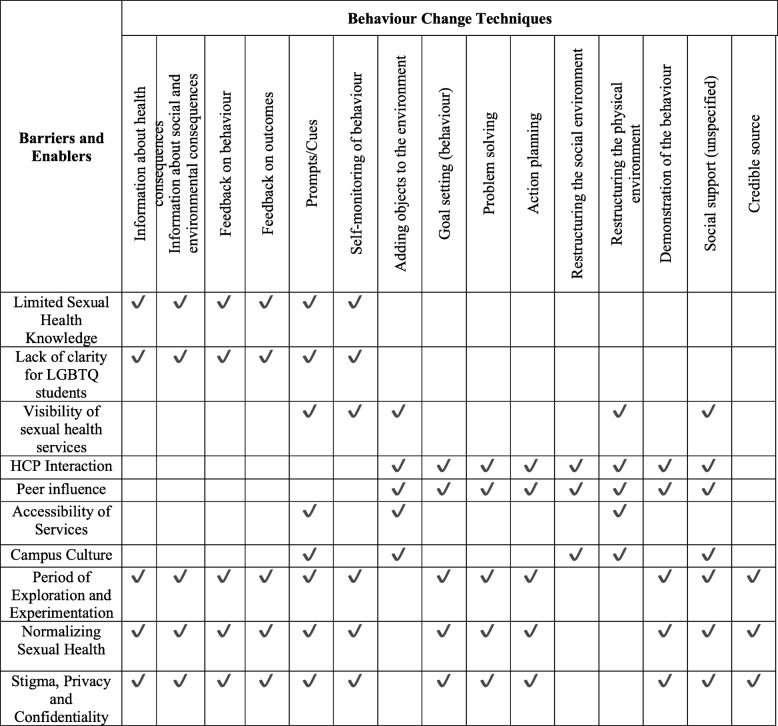
Barriers and enablers mapped to selected behaviour change techniques in the BCTTv1 [14]

Next, we used the BCW matrix of BCTs and intervention functions [12] to identify BCTs most likely to bring about change in students’ sexual health behaviours. From there, the research team used the APEASE criteria to narrow down this list and identified the following 15 BCTs as relevant to students’ use of sexual health services: *information about health consequences, information about social and environmental consequences, feedback on behaviour, feedback on outcome(s) of behaviour, prompts/cues, self-monitoring of behaviour, adding objects to the environment, goal setting (behaviour), problem solving, action planning, restructuring the social environment, restructuring the physical environment, demonstration of the behaviour, social support (unspecified)*, and *credible source* (Fig. 4). Lastly, the research team added their ideas to the list of potential modes of delivery for each BCT.

### Step 3: stakeholder consultation

We met with one key stakeholder at each university to discuss the study findings, brainstorm potential intervention modes of delivery, and prioritize BCTs that would be most feasible to implement at their university at the student, health care provider, and/or service level. The university stakeholders included a health services director and an advanced practice nurse who focuses on health promotion program planning. The stakeholders provided valuable contextual data on what BCTs and modes of delivery would be relevant for their context based on the resources available to them. The intervention modes of delivery and most feasible BCTs for implementation are outlined in Additional file 1.

#### Capability

To address the psychological capability barriers and enablers, we identified education, environmental restructuring, and enablement as appropriate intervention functions and the following nine BCTs: *information about health consequences, information about social and environmental consequences, feedback on behaviour, feedback on outcome(s) of behaviour, prompts/cues, self-monitoring of behaviour, adding objects to the environment, restructuring the physical environment, and social support (unspecified).* Potential modes of delivery include: education sessions during orientation week; emails and text messages with information about sexual health and sexual health services; and using Residence Assistants as key informants for sexual health.

#### Opportunity

To address the social and physical opportunity barriers and enablers, we found enablement, modelling, and environmental restructuring intervention functions to be most relevant. The following nine BCTs were identified: *prompts/cues, goal setting (behaviour), problem solving, action planning, restructuring the social environment, restructuring the physical environment, demonstration of the behaviour, adding objects to the environment,* and *social support (unspecified).* Potential modes of delivery include: mobile STI testing clinics; peer outreach; flexible hours of operation; and creating a friendly and welcoming space.

#### Motivation

Intervention functions to address the barriers and enablers under automatic and reflective motivation include: education, persuasion, modelling, enablement, and incentivisation. The following 12 BCTs were identified: *information about health consequences, information about social and environmental consequences, feedback on behaviour, feedback on outcome(s) of behaviour, prompts/cues, self-monitoring of behaviour, credible source, demonstration of the behaviour, social support (unspecified), goal setting (behaviour), problem solving,* and *action planning.* Potential modes of delivery include: peer support groups and student outreach; health care providers and students present during orientation; email or text message reminder of sexual health services and upcoming mobile clinics.

Following these three stages, we created a toolbox for our stakeholders to use in future sexual heath intervention design and program planning (Additional file 1). The behaviour change toolbox includes: the barriers and enablers to sexual health service use among university students under the COM-B components; six intervention functions most likely to bring about change; 15 BCTs to include as active ingredients in interventions; and a list of potential modes of intervention delivery. An electronic copy of the toolbox was sent to the participants of each stakeholder consultation meeting.

## Discussion

This study describes the systematic process of using the BCW to develop an intervention to improve university students’ use of sexual health services. We merged multiple data sources, including survey, focus group and interview data, to describe the barriers and enablers to sexual health service use among university students. Next, we mapped the barriers and enablers onto relevant intervention functions and BCTs to include as active ingredients in an intervention. We conducted stakeholder consensus meetings to narrow down the list to the most feasible and appropriate BCTs for the context of university students’ use of sexual health services and identified potential modes of intervention delivery.

### Behaviour change toolbox

The barriers and enablers to sexual health service use were similar for students at the two participating universities; however, we found differences in what intervention strategies would work best for each university due to differences in context and resources. As a result, we did not design one, all-encompassing intervention to implement at both universities. Instead, we met with key stakeholders from each university to identify BCTs that would be a priority for their school, and feasible modes of delivery based on the resources available to them. In the end, we developed a theory- and evidence-based toolbox of six intervention functions and 15 BCTs that can be used to design, implement and evaluate sexual health service interventions.

The toolbox presents many benefits for the health care providers and administrators involved in this study and decision-makers in similar settings. First, the toolbox provides a range of theory- and evidence-based resources for administrators in university health care settings to strengthen current services and plan for the delivery of future sexual health services. Second, many of the BCTs in the toolbox target three or more of the barriers and enablers to sexual health service use. The multi-targeted nature of these BCTs will be useful for stakeholders when advocating for funding for new sexual health programs: Administrators can demonstrate that by prioritizing these BCTs, they are able to address multiple barriers to sexual health service use. Third, the toolbox may also help to sustain theory- and evidence-based interventions at university health centres. Instead of providing the university with one intervention, we are presenting a variety of useful strategies that are malleable. Depending on the resources available, stakeholders can leverage existing structures (i.e., personnel, services, infrastructure) at their university to bring the BCTs to life. Lastly, the benefits of the toolbox extend beyond the two participating universities. Other universities may be able to use these theory- and evidence-based tools to develop interventions in their own context.

#### Behaviour change techniques

The theory- and evidence-based toolbox will likely be an improvement from the traditional atheoretical approach to intervention design in this context; however, the effectiveness of the six intervention functions and 15 BCTs to improve sexual health service use among university students is not yet known. Several studies have examined some of these BCTs in the context of sexual health services and found significant effects. Wolfers, de Zwart, and Kok [24] and Newby et al. [25] used intervention mapping [26] to design an intervention aimed at improving STI testing rates and increasing sexual health service uptake, respectively. These interventions include eight of the 15 BCTs identified in this study (*information about health consequences, information about emotional consequences, adding objects to the environment, feedback on outcomes of behaviour, social support (unspecified), information about health consequences, demonstration of behaviour, credible source*). Both interventions yielded significant positive results, including higher STI testing rates [24] and significant improvement in beliefs related to service access (i.e., service access being important and normal) among females, and a significant increase in the behaviour of visiting sexual health services among males [27]. The effectiveness of the BCTs used in these interventions shows promise for the similar BCTs identified in this current study. However, other than these few studies, the body of intervention literature on improving sexual health service use is scarce. Additional research is needed to test the effectiveness of the BCTs and intervention functions outlined in the toolbox.

#### Intervention functions

University health care providers and administrators can use the intervention functions described in the toolbox to translate the 15 BCTs into intervention content. Our results show that the *Education* intervention function maps onto five barriers and enablers to sexual health service use among university students. Studies have demonstrated that education interventions have moderate impact on sexual knowledge and attitudes [28, 29]. However, an increase in knowledge alone does not always lead to behaviour change [28]. It is important to use targeted, multi-component interventions to combine education with other key elements to maximize the potential for behaviour change [30, 31]. As such, it may be beneficial to target students with educational interventions that include multiple BCTs, such as *information about health consequences, information about social and environmental consequences, and demonstration of the behaviour*. University students may benefit from a sexual health education intervention that also includes the *prompts/cues* BCT in the form of electronic reminders. Our student participants recommended email and text message reminders to increase their awareness of sexual health services and the reasons to access them. Studies have shown that interventions delivered by mobile technologies increase the uptake of sexual health services and STI testing, particularly for tech-savvy young adults [32–34]. This is a widely available and accessible approach for university health centres to offer a confidential means of communicating sensitive or personal information with students [34]. Further, studies have found that using social media for sexual health education can help promote STI testing behaviours [35]. As such, there is an opportunity to leverage social media to support educational interventions that include BCTs aimed at increasing students’ capability and motivations for accessing sexual health services, such as *information about health consequences, information about social and environmental consequences, feedback on behaviour, prompts/cues, and self-monitoring of behaviour* [36].

The *Enablement* intervention function aligned with six barriers and enablers to students’ use of sexual health services. Enablement is described as “increasing means/reducing barriers to increase capability (beyond education and training) or opportunity (beyond environmental restructuring)” [12]. Several BCTs can be included in enablement interventions, such as *social support (unspecified), goal setting (behaviour), problem solving, action planning, adding objects to the environment, self-monitoring of behaviour, restructuring the physical environment*. Our stakeholders stated that enablement interventions related to a main priority at both universities: building capacity and resiliency among their student population. Strengthening students’ sexual resilience provides them with the tools needed to prevent negative outcomes from their sexual behaviour and take control of their physical, sexual, and mental health and well-being [37]. However, compared to education, enablement interventions have not been as extensively examined in the literature. Enablement interventions with the *social support (unspecified)* BCT are especially relevant in this context, as our behavioural analysis illustrated how peer influence can act as a barrier and enabler to sexual health service use. Studies have shown that perceived social norms affect sexual behaviours [38–40]. Young and Jordan [40] examined the influence of social networking photos on social norms and sexual health behaviours with a sample of college students in the United States. They found that students who viewed Facebook images with a low prevalence of sexually suggestive content estimated a larger percentage of peers used condoms and reported a greater intention to use condoms themselves in the future. In the context of university sexual health services, stakeholders could employ a similar approach with existing social media networks and curate positive images of peers accessing sexual health services to tap into students’ intentions for sexual health promotion behaviour.

### Utility of the Behaviours change wheel

The BCW offered a systematic approach for integrating multiple quantitative and qualitative data sources into the intervention design process. With its pragmatic, step-by-step framework, the BCW first helped to understand the range of factors influencing behaviour, all possible intervention options, and the full range of potential BCTs. As a result, we felt confident in choosing intervention content that was appropriate and relevant to the context of university sexual health service delivery. This study demonstrated the BCW’s utility for health researchers who do not have formal training in health psychology or behavioural science. The BCW made behaviour change theory tangible and pragmatic in the ‘real world’ of health services. Additional strengths and limitations to the utility of the BCW are described below.

#### Policy categories

The BCW includes seven broad policy categories to leverage behaviour change on a wider scale (e.g., changing legislation to encourage behaviour change at a population level) [12]. Similar to other intervention design researchers, the policy categories were found to be less practical than other BCW steps in this context [18, 41]. The selection of BCTs flowed logically from the COM-B model analysis and intervention functions. As such, we did not identify policy categories at this stage in the intervention development. Similar to Mc Sharry et al.’s recommendations [18], we believe that the policy categories will likely be more useful for broad, process-level guidance when designing implementation strategies for future sexual health service interventions.

#### Context

The influence of context on intervention effectiveness is often overlooked in the intervention design process, particularly when focusing on individual-level behaviours [42]. The BCW recommends gathering input from a diverse group of stakeholders to examine the influence of context at multiple conceptual levels. Moore and Evans [42] recommend using this co-production approach with stakeholders with intimate knowledge of the context. In this study, we included stakeholders at the barriers and enablers assessment stage, as well as the intervention design stage. This helped us to move from a theoretical exercise of listing intervention functions and BCTs to a hands-on approach with our stakeholders to address the question of “What is likely to work in this situation for these people in this organization with these constraints?“ [43]

We identified several barriers and enablers directly related to the social and physical context of sexual health behaviours on campus. From this, we identified several system-level BCTs, including *restructuring the social environment, restructuring the physical environment,* and *adding objects to the environment.* A limitation of the BCW is its lack of guidance on how contextual mechanisms function across different settings and its limited detail on the characteristics of system-level BCTs. Other researchers have had similar experiences in using the TDF to examine multi-level behavioural problems [15, 43–47]. To address this issue, some researchers have paired the TDF with organizational context frameworks, such as the Consolidated Framework for Implementation Research (CFIR), which elaborates on organizational-level determinants [48]. Future sexual health service intervention research would benefit from a similar approach to provide a more in-depth examination of the organizational context and how it influences service delivery. Similarly, we echo recent calls for future methodological research to elaborate upon system-level BCTs and characterise their meanings in more detail [49].

#### Reporting BCTs

Traditionally, behaviour change interventions are inadequately reported which hinders the reader’s ability to accurately understand, evaluate, or replicate interventions [50, 51]. When theory is used to describe the plausible mechanisms of action, findings can be synthesized with existing literature to inform future replication and evaluation studies [52]. Recent efforts to improve the implementation and replication of effective interventions have led to the development of reporting guidelines, such as the Template for Intervention Description and Replication (TIDieR) – a 12-item checklist that aims to standardize intervention descriptions [53]. Further, the BCTTv1 was developed to offer a shared language for clearly labelling and defining BCTs to ensure that behaviour change interventions are interpreted in the same way by different readers [14, 51]. The clear reporting of BCTs in this study will inform the science on sexual health behaviour change interventions. Researchers, administrators, and sexual health program planners can use the toolbox to identify intervention functions and BCTs that apply to their context and test them in implementation and evaluation studies. This will further aid in building a repository of effective sexual health service interventions and intervention components.

### Limitations

This final phase of our mixed methods study presents the following limitations. First, we followed the BCW steps closely, with the exception of the initial steps used to define and select the target behaviour. We had previously specified our target behaviour (sexual health service use among university students) through a literature review. In doing so, we may have missed a candidate behaviour that could impact students’ sexual health outcomes. Future research in this area would benefit from first defining the problem in behavioural terms and then selecting the target behaviour to ensure a rigorous and comprehensive approach to intervention design. Second, we found environmental context and resources to be an important barrier and enabler to sexual health service use. However, the BCW lacks clarity on what contextual parameters need to be in place for BCTs to be effective. Moving forward, it will be important to understand the contextual factors influencing BCT effectiveness. Lastly, due to scheduling conflicts, we were unable to conduct stakeholder meetings with students, health care providers, and administrators together. A joint meeting with all stakeholders may have led to different ideas and suggestions for intervention mode of delivery.

### Future research

The formative work described in this paper provides a strong foundation for future implementation and evaluation studies. We have clearly outlined proposed mechanisms of action that can be tested to build our understanding of what mechanisms work in the context of university sexual health care [54]. Further research is needed to identify implementation strategies for using the BCT toolbox in practice. It will be important to examine the conditions needed to support the use of the toolbox to design sexual health service interventions. Next steps include working with universities to examine these conditions and develop implementation strategies. This should include a clear roadmap for implementing the intervention functions and BCTs to maximize effectiveness and sustainability of the interventions. Furthermore, additional research is needed to evaluate the impact of providing stakeholders with a toolbox to design interventions that fit within their context, in comparison to a one-size-fits-all intervention. Lastly, efforts are needed to test the effect of different combinations of the six intervention functions and 15 BCTs on student health and health system outcomes.

## Conclusions

The BCW offered a systematic and pragmatic approach for intervention development and description. Following a detailed behavioural analysis, we used the BCW to identify six intervention functions and 15 BCTs to address the barriers and enablers to sexual health service use. These findings were packaged in a toolbox to provide users with theory- and evidence-based tools to design sexual health service interventions that meet the needs of their context. Future research is needed to test the utility of the toolbox for designing sexual health interventions and investigate the effectiveness of the BCTs and intervention functions outlined in the toolbox.

## Supplementary information

**Additional file 1.** Toolbox of intervention functions, behaviour change techniques, and modes of delivery.

## Data Availability

De-identified datasets analysed in this study are available from the corresponding author on reasonable request.

## Abbreviations

BCW: Behaviour Change Wheel
CO: Beliefs about Consequences
COM-B: Capability, Opportunity, Motivation and Behaviour
E: Environmental Context and Resources
EM: Emotion
FG: Focus group
HIV: Human immunodeficiency virus
K: Knowledge
LGBTQ: Lesbian, Gay, Bisexual, Transgender, Queer
MAD: Memory, Attention, and Decision-Making Processes
OP: Optimism
Pap: Papanicolaou
RA: Research assistant
SI: Social Influences
STI: Sexually transmitted infection
TDF: Theoretical Domains Frameworks
