# Supplementary material for: Designing an intervention to improve sexual health service use among university undergraduate students: a mixed methods study guided by the behaviour change wheel
Source: BMC Public Health. 2019 Dec 26;19:1734. doi: 10.1186/s12889-019-8059-4 (PMC6933635; doi:10.1186/s12889-019-8059-4)
Supplement: Supplementary file 1 — Additional file 1. Toolbox of intervention functions, behaviour change techniques, and modes of delivery. [file 12889_2019_8059_MOESM1_ESM.docx]

Additional file 1. Toolbox of intervention functions, behaviour change techniques, and modes of delivery.

| **COM-B** | **Barriers & Enablers to Sexual Health Service Use** | **Intervention Functions** | **Behaviour Change Techniques** | **BCT Definition^15^** | **Examples of Content** | **Examples of Mode of Delivery** |
| --- | --- | --- | --- | --- | --- | --- |
| **Capability**  **Motivation** | Limited sexual health knowledge and awareness  Lack of clarity for LGBTQ students  Period of exploration and experimentation  Normalizing sexual health  Stigma, privacy and confidentiality | Education  Environmental Restructuring  Persuasion  Modelling  Enablement  Incentivisation | *Information about health consequences^a,b^* | Provide information (e.g. written, verbal, visual) about health consequences of performing the behaviour | - Sexual health facts - Service Information - Short videos showing location, what an STI test look likes - Harm reduction content | - Presentations during orientation week - Residence Assistants as key informants - Facbeook, Instagram, Youtube - Email and text messages - Posters - Educational workshops with clinicians |
| **Capability**  **Motivation** | Limited sexual health knowledge and awareness  Lack of clarity for LGBTQ students  Period of exploration and experimentation  Normalizing sexual health  Stigma, privacy and confidentiality | Education  Environmental Restructuring  Persuasion  Modelling  Enablement  Incentivisation | *Information about social and environmental consequences ^a,b^* | Provide information (e.g. written, verbal, visual) about social and environmental consequences of performing the behaviour |  |  |
| **Capability**  **Motivation** | Limited sexual health knowledge and awareness  Lack of clarity for LGBTQ students  Period of exploration and experimentation  Normalizing sexual health  Stigma and feelings of shame | Education  Environmental Restructuring  Persuasion  Modelling  Enablement  Incentivisation | *Feedback on behaviour^a^* | Monitor and provide informative or  evaluative feedback on performance of the behaviour (e.g. form, frequency, duration, intensity) | - Previous and upcoming appointments - Upcoming mobile clinics (pop-up STI testing clinics offered across campus) | - Student health promotion outreach coordinator position - Use persuasive messaging in emails, posters, text messages |
| **Capability**  **Motivation** | Limited sexual health knowledge and awareness  Lack of clarity for LGBTQ students  Period of exploration and experimentation  Normalizing sexual health  Stigma, privacy and confidentiality | Education  Environmental Restructuring  Persuasion  Modelling  Enablement  Incentivisation | *Feedback on outcomes of behaviour* | Monitor and provide feedback on the outcome or performance of the behaviour |  |  |
| **Capability**  **Motivation** | Limited sexual health knowledge and awareness  Lack of clarity for LGBTQ students  Visibility of sexual health services  Accessibility of services  Campus culture  Period of exploration and experimentation  Normalizing sexual health  Stigma, privacy and confidentiality | Education  Environmental Restructuring  Persuasion  Modelling  Enablement  Incentivisation | *Prompts/Cues^a,b^* | Introduce or define environmental or social stimulus with the purpose of prompting or cueing the behaviour. The prompt or cue would normally occur at the time or place of performance. | - Text messages about previous and upcoming appointments - Sexual health facts      - Sexual health service information | - Social media - Text messages - Emails - Posters on campus |
| **Capability**  **Motivation** | Limited sexual health knowledge and awareness  Lack of clarity for LGBTQ students  Visibility of sexual health services  Period of exploration and experimentation  Normalizing sexual health  Stigma, privacy and confidentiality | Education  Environmental Restructuring  Persuasion  Modelling  Enablement  Incentivisation | *Self-monitoring of behaviour* | Establish a method for the person to  monitor and record their behaviour(s) as part of a behaviour change strategy | - Track of how often students attend the clinic - Making appointments online - Promote self-efficacy | - App or online platform - Nurse-patient relationships |
| **Capability**  **Opportunity** | Visibility of sexual health services  HCP Interaction  Peer influence  Accessibility of services  Campus culture | Education  Environmental Restructuring  Enablement   Modelling | *Adding objects to the environment^a^* | Add objects to the environment in order to facilitate performance of the behaviour | - Sexual health facts - Sexual health service information - Hours of operation that are flexible to students’ schedules | - Posters around campus - Recurring emails and text messages - Social media |
| **Opportunity**  **Motivation** | HCP Interaction  Peer influence  Period of exploration and experimentation  Normalizing sexual health  Stigma, privacy and confidentiality | Environmental Restructuring  Persuasion  Modelling  Enablement  Education  Incentivisation | *Goal setting (behaviour)* | Set or agree on a goal defined in terms of the behaviour to be achieved | - Nurses and students working together to build plan of care (e.g., coming to clinic after new sexual partners; creating goals for staying healthy) | - Nurse-student appointments |
| **Opportunity**  **Motivation** | HCP Interaction  Peer influence  Period of exploration and experimentation  Normalizing sexual health  Stigma, privacy and confidentiality | Environmental Restructuring  Persuasion  Modelling  Education  Enablement  Incentivisation | *Problem solving* | Analyze, or prompt the person to analyze, factors influencing the behaviour and generate or select strategies that include overcoming barriers and/or increasing facilitators | - Nurses and students working together at appointments to solve sexual health problems - At appointments, discuss how to address negative peer influence (e.g., with first year students) | - Nurse-student appointments |
| **Opportunity**  **Motivation** | HCP Interaction  Peer influence  Period of exploration and experimentation  Normalizing sexual health  Stigma, privacy and confidentiality | Environmental Restructuring  Persuasion  Modelling  Education  Enablement  Incentivisation | *Action planning* | Prompt detailed planning of performance of the behaviour (must include at least one of context, frequency, duration and intensity). Context may be environmental (physical or social) or internal (physical, emotional or cognitive) | - Promote scheduling appointments in advance to ensure clinician continuity - Plan schedule for year - Build capacity early in university journey | - Clinician-student appointments - Educational workshops during first year student orientation |
| **Opportunity** | HCP Interaction  Peer influence  Campus culture | Enablement  Modelling  Environmental Restructuring | *Restructuring the social environment* | Change, or advise to change the social environment in order to facilitate performance of the wanted behavior or create barriers to the unwanted behavior | - Friendly, welcoming space - Promoting peer support related to sexual health around residences and campus | - Health services’ physical environment - Residence assistants as key informants |
| **Opportunity** | Visibility of sexual health services  HCP Interaction  Peer influence  Accessibility of services  Campus culture | Enablement  Modelling  Environmental Restructuring | *Restructuring the physical environment* | Change, or advise to change the physical environment in order to facilitate performance of the wanted behaviour or create barriers to the unwanted behaviour | - Revise hours of operation - Longer appointment times - Keeping certain number of appointment times available for sexual health reasons | - Mobile clinics - Service policies |
| **Opportunity**  **Motivation** | Visibility of sexual health services  HCP Interaction  Peer influence  Period of exploration and experimentation  Normalizing sexual health  Stigma, privacy and confidentiality | Enablement  Modelling  Environmental Restructuring  Education  Persuasion  Incentivisation | *Demonstration of the behaviour* | Provide an observable sample of the performance of the behaviour, directly in person or indirectly e.g. via film, pictures, for the person to aspire to or imitate | - “How to” access service videos and information - Building rapport with students in sexual health setting - Student Outreach | - YouTube videos, Facebook and Instagram - Clinician workshops - Residence Assistants |
| **Opportunity**  **Motivation** | Visibility of sexual health services  HCP Interaction  Peer influence  Campus culture    Period of exploration and experimentation  Normalizing sexual health  Stigma, privacy and confidentiality | Enablement  Modelling  Environmental Restructuring  Education  Persuasion  Incentivisation | *Social Support (unspecified)^a,b^* | Advise on, arrange or provide social support (e.g. from friends, relatives, colleagues,’ buddies’ or staff) or non-contingent praise or reward for performance of the behavior. It includes encouragement and counselling, but only when it is directed at the behavior | - Sexual health counselling - Student Outreach | - Nurse counselling sessions - Peer support groups - Residence Assistants |
| **Motivation** | Period of exploration and experimentation  Normalizing sexual health  Stigma, privacy and confidentiality | Education  Persuasion  Incentivisation  Modelling  Enablement | *Credible Source* | Present verbal or visual communication from a credible source in favour of or against the behavior | - Sexual health facts - Sexual health service information | - Residence Assistants - Having clinicians or students give presentations during orientation - Partner with Nursing, Medicine, Allied Health and Health Promotion students |

^a^ = prioritized BCTs by stakeholders at University A as most feasible to implement

^b^ = prioritized BCTs by stakeholders at University B as most feasible to implement
